# Impact of metabolic comorbidities on the clinical course of West Nile virus infection

**DOI:** 10.1007/s40618-026-02871-x

**Published:** 2026-03-26

**Authors:** G. Guarisco, I. Milani, F. Leonetti, D. Capoccia, M. Chinucci, G. Mancarella, R. Marocco, M. Lichtner, C. Del Borgo, A. Parente

**Affiliations:** 1 Department of Medical-Surgical Sciences and Biotechnologies, Sapienza University of Rome, Rome, Italy; 2 Infectious Diseases Unit, SM Goretti Hospital, Sapienza University of Rome, Latina, Italy; 3 Infectious Disease Unit, Sant’Andrea Hospital, Sapienza University of Rome, Rome, Italy; 4 Department of Neuroscience, Mental Health, and Sense Organs, NESMOS, Sapienza University of Rome, Rome, Italy

**Keywords:** Infectious, Diabetes mellitus, Metabolic status

## Abstract

**Background:**

Italy has experienced a marked increase in the incidence of human infections caused by West Nile virus (WNV). Metabolic and other comorbidities, particularly hyperglycemia, are associated with unfavorable outcomes in infectious diseases. This study aimed to investigate the association between metabolic comorbidities, with a specific focus on blood glucose (BG) levels at hospital admission, and the clinical course of WNV infection.

**Methods:**

34 patients (22 males, 12 females, mean age 69 ± 14 years) with confirmed WNV infection were included. Clinical history, metabolic comorbidities, anthropometric and biochemical parameters at admission, and outcomes were collected.

**Results:**

Arterial hypertension (35.3%), dyslipidemia (23.5%), and diabetes mellitus (8.8%) were highly represented metabolic comorbidities. 35.3% of patients had two comorbidities and 32.4% had three comorbidities, all of which included at least one of the metabolic comorbidities, while more than half had fasting BG levels ≥ 100 mg/dL or fasting BG levels > 125 mg/dL at admission. Duration of hospital stay increased significantly with BG category, with more days observed in patients with BG ≥ 100 mg/dL, whereas no significant differences were observed between Body Mass Index (BMI) groups. Correlation analyses showed a positive association of BG levels with days of hospitalization and Cerebrospinal fluid (CSF) glucose concentration. Patients who died had at least two comorbidities and BG levels > 125 mg/dL at admission.

**Conclusion:**

These results highlight the importance of BG levels at admission as a prognostic factor in WNV infection. Early recognition and management of elevated BG levels may be an important adjunct in the clinical care of affected patients.

## Introduction

In recent months, Italy has experienced a marked increase in the incidence of human infections caused by West Nile virus (WNV), a mosquito-borne flavivirus endemic in several European countries that cause recurrent epidemics of meningitis and encephalitis in humans and horses [1]. Since October 2025, surveillance data from the *Istituto Superiore di Sanità (ISS)* reported 718 laboratory-confirmed cases, including 341 cases of West Nile neuroinvasive disease (WNND) and 49 deaths [2]. One of the most affected regions was Lazio, particularly the province of Latina [3, 4], a swampy area reclaimed a century ago.

WNV infection manifests with a wide clinical spectrum, ranging from asymptomatic or mild febrile illness to severe neuroinvasive disease with a high risk of mortality and long-term neurological sequelae. There is increasing evidence that metabolic comorbidities, particularly hyperglycemia, are associated with adverse outcomes in infectious diseases, independent of a pre-existing diagnosis of diabetes mellitus, as demonstrated during the COVID-19 pandemic [5, 6]. Recent studies have suggested that cardiovascular disease, stroke, diabetes mellitus, respiratory disease, and renal disease may contribute to the severe clinical manifestations of WNV [7, 8]. In particular, diabetes mellitus, has been recognized as a risk factor for severe clinical manifestations and death from the disease, suggesting that early detection and appropriate management strategies are critical to reducing morbidity and mortality in patients with diabetes mellitus [9]. Thus, glycemic control would be an alternative tool to improve the outcome of some infections even in patients without diabetes mellitus [10].

However, specific studies analyzing the relationship between diabetes mellitus and the severity of WNV infection are severely limited, preventing the accurate identification of individuals at greatest risk and the development of targeted prevention strategies at the national level.

This study aimed to investigate the association between metabolic comorbidities, with a particular focus on blood glucose (BG) levels at hospital admission, and the clinical course of WNV infection. We retrospectively analyzed cases admitted to *Santa Maria Goretti Hospital* in Latina (Polo Pontino, Sapienza University of Rome) between July 9 and August 20, 2025.

## Methods

A retrospective analysis of medical records was performed on patients with WNV infection admitted to the *Santa Maria Goretti Hospital* in Latina (Polo Pontino, Sapienza University of Rome) between July 9 and August 20, 2025.

Clinical history, metabolic and other comorbidities, biochemical parameters at admission, and outcomes were collected. Body Mass Index (BMI) was calculated as weight [kg]/(height [m])^2^. Metabolic comorbidities were defined according to the components of the metabolic syndrome and included hyperglycemia (or previously diagnosed diabetes mellitus, obesity, dyslipidemia and arterial hypertension [11].

According to on World Health Organization guidelines, patients were stratified into three categories based on BMI:

- < 25 kg/m2 (normal-weight);
- 25–29.9 kg/m2 (overweight);
- ≥ 30 kg/m2 (obesity).

Patients were stratified into three groups according to on the fasting BG value measured with a single blood sample on the morning of the first day of hospitalization:

- BG < 100 mg/dL (Group 1).
- BG 100–125 mg/dL (Group 2).
- BG > 125 mg/dL (Group 3).

Continuous variables were expressed as mean and standard deviation or median and Interquartile range (IQR) according to their distribution. Categorical variables were recorded as frequencies and percentages. Mann-Whitney test and Kruskal-Wallis test were used to assess differences among groups and post hoc, p-value were adjusted according to Benjamini to account for multiple comparisons. Spearman’s correlation coefficient was used to assess relationship between BG, days of hospitalization and CSF glucose. P-value < 0.05 was considered as statistically significant. Analyses were performed using SPSS version 21.

## Results

A total of 34 patients (22 males, 12 females, mean age 69 ± 14 years) with confirmed WNV infection were included. Highly represented metabolic diseases were arterial hypertension (35.3%), dyslipidemia (23.5%), and diabetes mellitus (8.8%). A total of 55.8% of patients were classified as normal-weight, 44.1% as having overweight/obesity, 47% of patients had BG < 100 mg/dL, while a total of 53% of patients had BG ≥ 100 mg /dL (Table 1). Multiple comorbidities were frequent, with two (35.3%) and three (32.4%) comorbidities, including at least one metabolic comorbidity (hyperglicemia - BG ≥ 100 mg/dl -, diabetes mellitus, obesity, dyslipidemia, or hypertension).

**Table 1:** Comorbidities in the study population (N = 34).

Comorbidities	*n* (%)
Diabetes mellitus*	3/34 (8,8%)
OVERWEIGHT/obesity*	15/34 (44,1%)
DyslipidemiA*	8/34 (23,5%)
Hypertension*	12/34 (35,3%)
Ischemic heart disease	6/34 (17,6%)
Ictus	3/34 (8,8%)
Heart failure	1/34 (2,9%)
Chronic kidney disease	1/34 (2,9%)
Kidney transplantation	1/34 (2,9%)
Arrhythmia with or without pacemaker implantation	5/34 (14,3%)
Other heart diseases	4/34 (11,8%)
Oncological diseases	4/34 (11,8%)
Chronic obstructive pulmonary disease	1/34 (2,9%)
Rheumatoid arthritis	1/34 (2,9%)
Lupus nephritis	1/34 (2,9%)
Major depression	1/34 (2,9%)
Gastroesophageal reflux disease	1/34 (2,9%)
Deep vein thrombosis	1/34 (2,9%)
Benign prostatic hypertrophy	2/34 (5,9%)
3 comorbidities^§^	11/34 (32,4%)
2 comorbidities^§^	12/34 (35,3%)
1 comorbidity	3/34 (8,8%)
No comorbidities	8/34 (23.5%)
BG 100–125*	9/34 (26,5%)
Bg > 125*	9/34 (26,5%)

BG: Blood glucose; *metabolic comorbidities; ^§^ of which at least one is a metabolic comorbidity

**Fig. 1:**
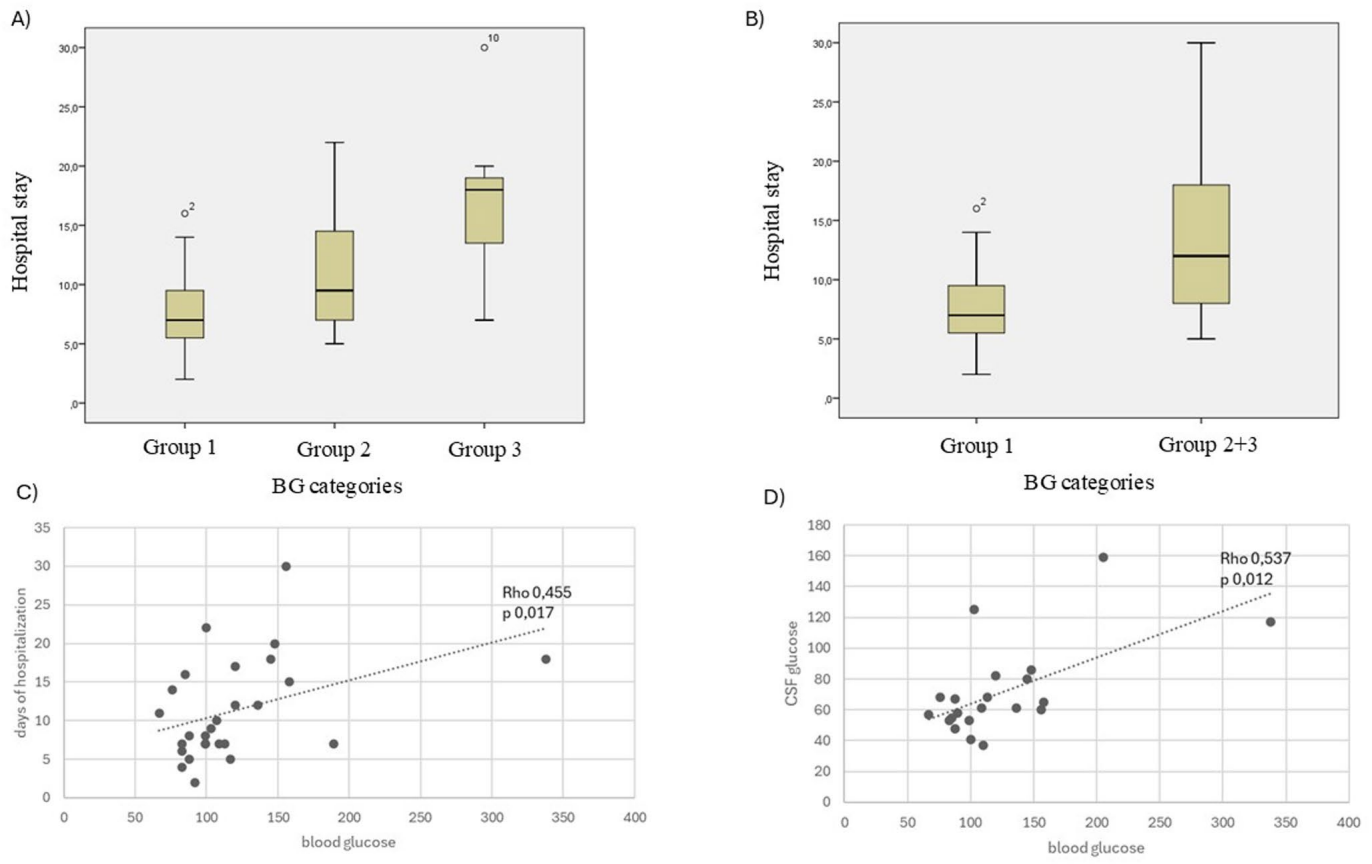
Duration of hospitalization by BG category (**A-B**) and linear regression analysis between BG and hospital stay (**C**) and between CSF glucose and blood glucose (**D**). BG: Blood glucose. Figure 1A shows the duration of hospitalization for blood glucose < 100 mg/dl (Group 1), 100–125 mg/dl (Group 2) and > 125 mg/dl (Group 3). Figure 1B shows the comparison of duration of hospital stay between patients with BG < 100 mg/dL (Group 1) and all patients with BG ≥ 100 mg/dL (Group 2 + 3). Figure 1C shows linear regression analysis between BG and hospital stay. Figure 1D shows linear regression analysis between CSF glucose and blood glucose

Median hospital stay was 9 days (IQR 7–16). Duration of hospitalization varied significantly by BG category (Fig. 1A):

- Group 1 (BG < 100 mg/dL): 7 days (IQR 5–10).
- Group 2 (BG 100–125 mg/dL): 9 days (IQR 7–15).
- Group 3 (BG > 125 mg/dL): 18 days (IQR 12–20).

The difference between Group 1 e 3 was statistically significant (*p* = 0.004). When comparing patients with BG < 100 mg/dL (Group1) with all patients presenting with BG ≥ 100 mg/dL (Group 2 + 3) (Fig. 1B**)**, hospitalization was significantly longer in the latter (12 [IQR 7–18] vs. 7 [IQR 5–10] days, *p* = 0.014).

According to BMI, median hospital stay was:

- Normal-weight: 10 (8,4-15,7) days.
- Overweight: 7 (3,6-13,2) days.
- Obesity: 14 (3,3–23,4) days.

No significant differences were observed between BMI groups and length of hospital stay (*p* = 0.25).

Correlation analyses showed that BG levels were positively associated with both days of hospitalization (rho = 0.455, *p* = 0.017) and CSF glucose concentration (rho = 0.537, *p* = 0.012) (Fig. 1C and D).

Three patients died, all males aged 77, 80, and 86 years. Each presented at least two metabolic abnormalities according to our study definition, and all exhibited blood glucose levels > 125 mg/dL at admission (145, 148, and 338 mg/dL, respectively). Only one patient had a prior diagnosis of diabetes mellitus and hypertension, whereas the other two had no previously documented metabolic comorbidities before admission.

## Discussion

Our findings suggest that metabolic comorbidities are widely represented in people with WNV infection, with arterial hypertension, dyslipidemia, and diabetes mellitus being among the most common. Moreover, in patients with 2 or 3 comorbidities, at least one is a metabolic disease.

The diagnosis of obesity is still based on body mass index (BMI) cutoffs, thus failing to reflect the distribution of adipose tissue and not correlating with the degree of systemic inflammation and disease severity [12]. This could explain the absence of correlation between BMI and hospital stay days in our sample.

There is evidence recognizing that the presence of metabolic comorbidities can impair the immune system, leading to higher levels and prolonged duration of viremia [7]. Metabolic comorbidities have been associated with an increased likelihood of developing neuroinvasive disease [12], likely due to interference with immune system function and facilitation of viral entry through the blood-brain barrier [13]. Indeed, endothelial dysfunction, attenuation of anti-inflammatory responses and generation of a pro-inflammatory state are common features of both metabolic comorbidities and many infectious disorders [7].

More than half of the patients presented with fasting BG levels ≥ 100 mg/dL at admission, regardless of a known diagnosis of diabetes mellitus. Notably, we found an association between elevated blood glucose levels (≥ 100 mg/dL) and prolonged hospitalization in WNV infection as well as, with cerebrospinal fluid glucose concentration.

Cerebrospinal fluid (CSF) glucose is known to correlate with blood glucose levels, and our results confirm this [15]. It has also been shown that glucose bioenergetics in WNV infection is critical for viral replication, and that removing glucose from specific culture media reduced virus multiplication, as assessed by a plaque assay of viral yield [15]. Therefore, the correlation between blood glucose and CSF glucose could at least partially explain the longer length of hospital stay in patients with higher fasting glucose levels.

According to a recent meta-analysis, patients with WNV infection have an imbalance in glucose homeostasis within the central nervous system [14]. In particular, diabetes mellitus has been associated with an altered immune response that may allow further viral invasion, leading to greater susceptibility to severe forms of WNV and higher mortality [9]. For example, increased viral replication in the periphery and brain and higher post-infection mortality have been reported in *db/db* mice following WNV infection, correlating with dysfunction of antiviral immunity, delayed production of type I interferon (IFN-α), and reduced specific antibody responses (IgM and IgG) to the virus [16]. In experimental models, inhibition of glycolysis in monocyte cells has been shown to confer protection against WNV encephalitis by reducing the production of inflammatory cytokines, without affecting viral load [16, 17]. 

Consistent with our findings, a meta-analysis has also confirmed hypertension and diabetes mellitus as the most important metabolic comorbidities associated with WNV infection, especially in severe cases [7, 18, 19], and increased risk of mortality [8]. Specifically, the presence of diabetes mellitus was associated with worse infection outcomes, with an estimated odds ratio of approximately 2.2 for developing severe disease [9].

Our findings add to the evidence highlighting the critical role of glycolytic metabolism in modulating the pathological immune response in the brain [16], and suggest the glycolytic pathway as a promising target for future therapeutic intervention [14]. In addition, the prevalence of metabolic comorbidities in WNV patients highlights the need for further evaluation of their coexistence with infection. This could enable healthcare providers to better predict complications based on the presence of these comorbidities and to implement appropriate treatments.

Limitations of this study include the retrospective study design and small sample size. In addition, reliance on BMI as a measure of obesity has significant limitations because it does not reflect differences in body composition, such as muscle mass or fat distribution, which are important in assessing an individual’s metabolic health. A more accurate understanding could be achieved by incorporating measures by supplementing BMI with waist circumference measurements.

In addition, BG level, used to stratify our population, was measured only once and HbA1c was not available to assess glucose metabolism.

## Conclusion

To our knowledge, this is the first study to examine the impact of blood glucose levels on WNV infection and duration of hospitalization. Although limited by the small sample size, these observations highlight a potential importance of glycemic status as a prognostic factor in WNV infection and may guide clinicians to evaluate the presence of metabolic comorbidities and their involvement in infection severity. In particular, early recognition and management of elevated blood glucose levels may be an important adjunct in the clinical care of affected patients.

## References

[1] Gould CV, Staples JE, Guagliardo SAJ, Martin SW, Lyons S, Hills SL et al (2025) West Nile Virus: Rev JAMA 19 agosto 334(7):618–628 10.1001/jama.2025.8737 40622703

[2] Istituto Superiore di Sanità (2025) Sorveglianza integrata del West Nile e Usutu virus [Internet]. Epicentro; Disponibile su: https://www.epicentro.iss.it/westnile/bollettino/Bollettino_WND_2025_12.pdf

[3] European Centre for Disease Prevention and Control (ECDC) (2025) Authority (EFSA) EFS. Surveillance of West Nile virus infections in humans and animals in Europe, monthly report – data submitted up to 5 November 2025. EFSA J 23(11):e9762 41246254 10.2903/j.efsa.2025.9762 PMC12616260

[4] Mussetto I, Bongiovanni A, Colavita F, Giambi C, Sala MG, Del Borgo C et al (2025) Outbreak of autochthonous West Nile virus infection in Lazio region, Italy, July to August 2025: preliminary investigation. Euro Surveill 4 settembre 30(35):2500634 10.2807/1560-7917.ES.2025.30.35.2500634 PMC12413602 40910225

[5] Holt RIG, Cockram CS, Ma RCW, Luk AOY (2024) Diabetes and infection: review of the epidemiology, mechanisms and principles of treatment. Diabetologia 67(7):1168–1180 38374451 10.1007/s00125-024-06102-x PMC11153295

[6] Guarisco G, Fasolo M, Capoccia D, Morsello G, Carraro A, Zuccalà P et al (2022) Blood glucose and epicardial adipose tissue at the hospital admission as possible predictors for COVID-19 severity. Endocr 1 gennaio 75(1):10–18 10.1007/s12020-021-02925-5 PMC8563297 34729688

[7] Badawi A, Velummailum R, Ryoo SG, Senthinathan A, Yaghoubi S, Vasileva D et al (2018) Prevalence of chronic comorbidities in dengue fever and West Nile virus: A systematic review and meta-analysis. PLOS ONE. 10 luglio 13(7):e0200200 10.1371/journal.pone.0200200 PMC6039036 29990356

[8] Roberts JA, Kim CY, Hwang SA, Hassan A, Covington E, Heydari K et al (2025) Clinical, Prognostic, and Longitudinal Functional and Neuropsychological Features of West Nile Virus Neuroinvasive Disease in the United States: A Systematic Review and Meta-Analysis. Ann Neurol 98(1):93–106 40008684 10.1002/ana.27220

[9] Lu HZ, Xie YZ, Gao C, Wang Y, Liu TT, Wu XZ et al (2024) Diabetes mellitus as a risk factor for severe dengue fever and West Nile fever: A meta-analysis. PLOS Neglected Tropical Diseases. 31 maggio 18(5):e0012217 10.1371/journal.pntd.0012217 PMC11168630 38820529

[10] Chávez-Reyes J, Escárcega-González CE, Chavira-Suárez E, León-Buitimea A, Vázquez-León P, Morones-Ramírez JR et al (2021) Susceptibility for Some Infectious Diseases in Patients With Diabetes: The Key Role of Glycemia. Front Public Health 16 febbraio 9:559595 10.3389/fpubh.2021.559595 PMC7921169 33665182

[11] Doan TTA, Nguyen TS, Tran VK, Hoang NTD, Le TD, Nguyen ATD et al (2025) Prevalence and factors associated with metabolic syndrome and its components among overweight and obese reproductive-age women in Bac Giang, Vietnam. Sci Rep 25 novembre 15(1):42765 10.1038/s41598-025-27095-6 PMC12663542 41291061

[12] Frühbeck G, Busetto L, Dicker D, Yumuk V, Goossens GH, Hebebrand J et al (2019) The ABCD of Obesity: An EASO Position Statement on a Diagnostic Term with Clinical and Scientific Implications. Obes Facts maggio 12(2):131–136 10.1159/000497124 PMC6547280 30844811

[13] Sutinen J, Fell DB, Sander B, Kulkarni MA (2022) Comorbid conditions as risk factors for West Nile neuroinvasive disease in Ontario, Canada: a population-based cohort study. Epidemiol Infect gennaio 150:e103 10.1017/S0950268822000887 PMC9171902 35543409

[14] Van Dyken P, Lacoste B Impact of Metabolic Syndrome on Neuroinflammation and the Blood–Brain Barrier. Front Neurosci [Internet]. 11 dicembre 2018 [citato 8 ottobre 2025];12. Disponibile su: https://www.frontiersin.org/journals/neuroscience/articles/10.3389/fnins.2018.00930/full 10.3389/fnins.2018.00930 PMC6297847 30618559

[15] Mingo-Casas P, Blázquez AB, Gómez de Cedrón M, San-Félix A, Molina S, Escribano-Romero E et al (2023) Glycolytic shift during West Nile virus infection provides new therapeutic opportunities. J Neuroinflammation 27 settembre 20:217 10.1186/s12974-023-02899-3 PMC10537838 37759218

[16] Kumar M, Roe K, Nerurkar PV, Namekar M, Orillo B, Verma S et al (2012) Impaired Virus Clearance, Compromised Immune Response and Increased Mortality in Type 2 Diabetic Mice Infected with West Nile Virus. PLOS ONE 31 agosto 7(8):e44682 10.1371/journal.pone.0044682 PMC3432127 22953001

[17] Wishart CL, Spiteri AG, Tan J, Macia L, King NJC (2025) Metabolic Programming Drives Protective and Inflammatory Monocyte Fates in Viral Encephalitis. Adv Sci (Weinh) 14 luglio 12(33):e05844 10.1002/advs.202505844 PMC12412471 40657754

[18] Santini M, Haberle S, Židovec-Lepej S, Savić V, Kusulja M, Papić N et al (2022) Severe West Nile Virus Neuroinvasive Disease: Clinical Characteristics, Short- and Long-Term Outcomes. Pathogens gennaio 11(1):52 10.3390/pathogens11010052 PMC8779330 35056000

[19] Nikolić N, Milošević B, Miloš S, Mila L, Milošević I, Mitrović N et al (2025) Long-Term Follow-Up of Patients with West Nile Neuroinvasive Disease. Viruses 23 giugno 17(7):878 10.3390/v17070878 PMC12300566 40733496

